# Saliva as a source of reagent to study human susceptibility to avian influenza H7N9 virus infection

**DOI:** 10.1038/s41426-018-0160-8

**Published:** 2018-09-19

**Authors:** Ming Tan, Lunbiao Cui, Xiang Huo, Ming Xia, Fengjuan Shi, Xiaoyan Zeng, Pengwei Huang, Weiming Zhong, Weiwei Li, Ke Xu, Liling Chen, Minghao Zhou, Xi Jiang

**Affiliations:** 1 Division of Infectious Diseases, Cincinnati Children’s Hospital Medical Center, Cincinnati, OH USA; 2 Department of Pediatrics, University of Cincinnati College of Medicine, Cincinnati, OH USA; 3 Jiangsu Provincial Center for Disease Control and Prevention, Nanjing, Jiangsu Province China; 4 Suzhou Center for Disease Control and Prevention, Suzhou, Jiangsu Province China

## Abstract

Avian influenza H7N9 viruses are an important public health concern due to their high mortality rate and potentials for future pandemics. We investigated human susceptibility to H7N9 viruses using recombinant H7N9 hemagglutinin (HA) proteins as a probe and found a strong association between H7N9 infections and HA binding among saliva samples from 32 patients and 60 uninfected controls in Jiangsu province, China, during the 2016 epidemic season. We also found that sialyl Le^x^ (SLe^x^) antigen that was recognized by H7N9 HA was associated with H7N9 virus infection. Further analysis suggested that additional saccharide residues adjacent to the SLe^x^ moiety may affect the H7N9-binding specificity. Our data suggested that saliva may be a useful reagent to study human susceptibility to avian influenza H7N9 virus, which may impact the disease control and prevention of avian influenza viruses as important human pathogens.

## Introduction

Influenza A viruses (IAVs) are enveloped RNA viruses in the family Orthomyxoviridae. They exhibit a broad host range infecting both humans and various animal species, including wild birds, poultry, pigs, dogs, cats, horses, mink, bats, and marine mammals, among which waterfowl are believed to be IAV reservoirs. According to epidemiology and sources of virus origins, human flus can be sorted into several types, including seasonal flu that spreads rapidly and widely within human populations without involvement of specific animal vectors, avian flu that is transmitted from birds/chicken, and swine flu that is communicated from domestic pigs. Avian influenza viruses mainly circulate in wild birds and some of them infect chicken causing large outbreaks in chicken farms, leading significant economic loss. A few highly pathogenic avian influenza (HPAI) virus types, such as the HPAI H5N1 and HPAI H7N9 viruses, cause human infections with high mortality rates, while HPAI H7N3 viruses cause only mild disease in humans. The recently emerged H7N9 avian flu viruses in 2013 in China have caused five epidemic waves, infecting totally 1344 people with 511 deaths in China^1^, becoming a new public health threat.

The host ranges or host specificity of IAVs are believed to be determined by their host receptors that are a group of sialic acid (SA)-containing glycans with host species-specific SA-galactose (Gal) linkages, although non-SA-binding IAVs have been found in bats^2,3^. Human IAVs interact preferentially with SAs linked to Gal by α 2,6-linkage, whereas avian IAVs favor SA-α2,3-Gal. In addition, the relative abundance of two specific SA species, *N*-acetylneuraminic acid (NeuAc) or *N*-glycolylneuraminic acid (NeuGc), among different animal species could also affect the infection and replicative potential of IAVs^4^.

However, in addition to the α2,3- vs. α2,6-linkage SAs, other saccharide residue(s) may also affect the binding affinity and/or specificity of avian influenza viruses and thus might play a role in the host specificity of avian influenza viruses between humans and avian. For example, several IAVs isolated from terrestrial poultry were found to bind preferentially to 6-sulfo sialyl Lewis X or Su-SLe^x^ [SAα2,3-Galβ1,4 (Fucα1,3) (6HSO3)] and SLe^x^ antigens^5^. Most of the tested poultry IAVs, including those containing H5, H6, H7, and H9, shared high binding affinity to the Su-SLe^x^ and SLe^x^ antigens, implying that these two SLe^x^ are potential common receptors recognized by H5, H6, H7, and H9 IAVs of terrestrial poultry^5^. Similarly, a chicken IAV was reported to recognize fucosylated α2,3 sialoglycan (SLe^x^) receptors on the epithelial cells lining upper respiratory tracts of chickens^6^. The SA-binding profiles and structural bases of several human-infecting H7N9 2013 isolates have been studied; however, clear association of the presence/absence of the mammalian- vs. avian-signature residue Leu226 and binding of human vs. avian SA receptor analogs were not found^7^. Thus, the molecular basis or mechanism why and how the H7N9 and other avian IAVs emerged as important human pathogens remains elusive.

In this study, we characterized variations of human susceptibility to H7N9 IAV infections by testing saliva samples. Saliva samples have been widely used to study pathogen–host interaction to assess host susceptibility to infections of noroviruses and rotaviruses, which is associated with the host histo-blood group antigens (HBGAs) and SA-related carbohydrates^8–12^. Using recombinant H7 hemagglutinin (HA) as a probe, we found a strong association between H7 HA-saliva-binding signals and H7N9 virus infection among saliva samples from patients and uninfected controls. Further HBGA and sialyl glycan phenotyping of the saliva samples showed that the SLe^x^ and SA are important H7N9 HA-binding components. However, our data further suggested that additional unknown saccharide residues also affect H7 HA-glycan-binding specificity. Our finding highlighted saliva as a convenient source of reagent to study human susceptibility to avian influenza H7N9 virus infection, which would facilitate disease control and prevention of avian influenza viruses.

## Results

### Production and validation of functional H7 HA1 protein

**Fig. 1:**
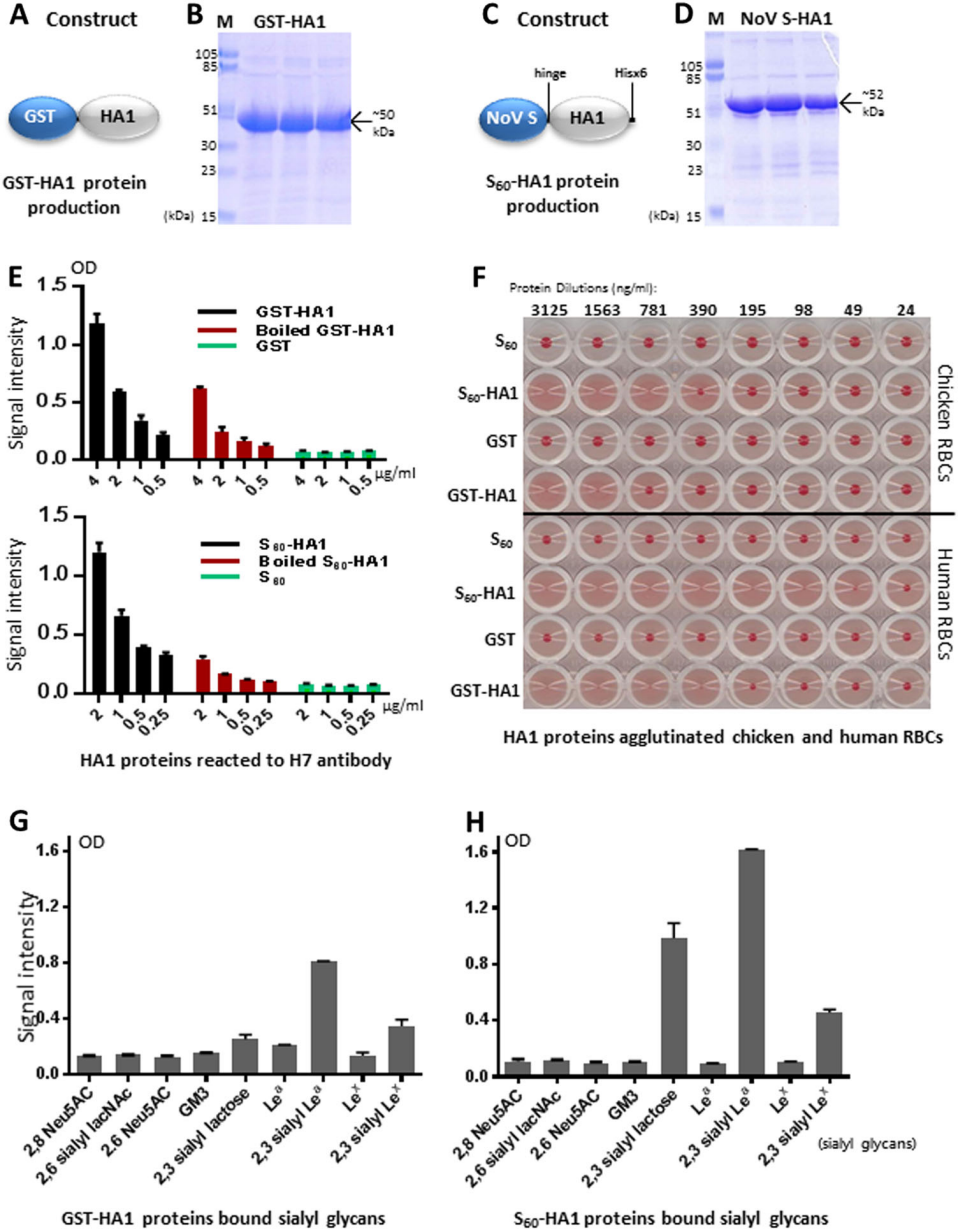
Production and validation of functional H7 HA1 protein. **a**–**d** The H7 HA1 proteins in forms of GST-HA1 fusion proteins (**a**, **b**) and S_60_-HA1 particles (**c**, **d**) were produced in *E. coli*. Their expression constructs are schematically illustrated in **a** (GST-fusion protein) and **c** (norovirus S domain fusion protein), while the expressed and purified proteins were analyzed by SDS-PAGE, respectively (**b**, **d**). Lane M is the prestained protein markers with indicated sizes. The remaining three lanes are three purified protein fractions that eluted from the affinity column. **e** Both GST-HA1 (upper panel, black) and S_60_-HA1 (lower panel, black) proteins reacted with H7-specific antibody, while the GST and S_60_ particles (negative controls, green) did not. After heat-inactivation both HA1 proteins reduced the reactivity to the H7 antibody. **f** Both S_60_-HA1 and GST-HA1 proteins agglutinated chicken (upper panel) and human (lower panel) red blood cells (RBCs). S_60_ particle and GST without HA1 were used as negative controls that did not agglutinate RBCs. The serially diluted protein concentrations are indicated. **g**, **h** Both GST-HA1 (**g**) and S_60_-HA1 (**h**) proteins bound certain sialyl glycans. *X*-axis, various sialyl glycans in the indicated names. *Y*-axis, binding signal intensity of the H7 HA1 proteins to various sialyl glycans. Error bars are standard deviations that are calculated by the software GraphPad Prism 6

The H7 HA1 protein in forms of GST-HA1 fusion proteins (Fig. 1a, b) and S_60_-HA1 particles (Fig. 1c, d) were produced (see Materials and methods for details) as soluble proteins at yields of 7–10 mg/liter bacterial culture. While a previous study showed that *Escherichia coli*-expressed IAV HA1 protein folds into its native structure with an intact receptor-binding site^13^, we further demonstrated here that both GST- and S_60_-HA1 fusion proteins reacted with IAV H7-specific antibody (LifeSpan BioSciences, Inc.) strongly by enzyme immunoassays (EIA) (Fig. 1e) and agglutinated human and chicken red blood cells (RBCs) with higher hemagglutination activity of the S_60_-HA1 than that of GST-HA1 (Fig. 1f). The heat-inactivated HA1 fusion proteins reduced their reactivity to the H7 antibody significantly, suggesting the loss of the conformational epitopes via the heat treatment (Fig. 1e). We then showed that the two HA1 proteins bound α2,3-, but not α2,6- or α2,8-linked sialyl glycans (Fig. 1g, h), consistent with the results reported by others^7,14,15^. These data validated the structural and functional integrity of our HA1 protein. We noted that the S_60_-HA1 protein gave higher and cleaner binding signals than those of the GST-HA1 fusion protein (Fig. 1, compared g with h), likely due to the high avidity of the S_60_-HA1 particles compared with the bivalent GST-HA1 protein. Thus, the S_60_-HA1 proteins were used for the downstream saliva-binding experiments.

### H7 HA1-saliva-binding signals were associated with H7N9 virus infection in humans

**Fig. 2:**
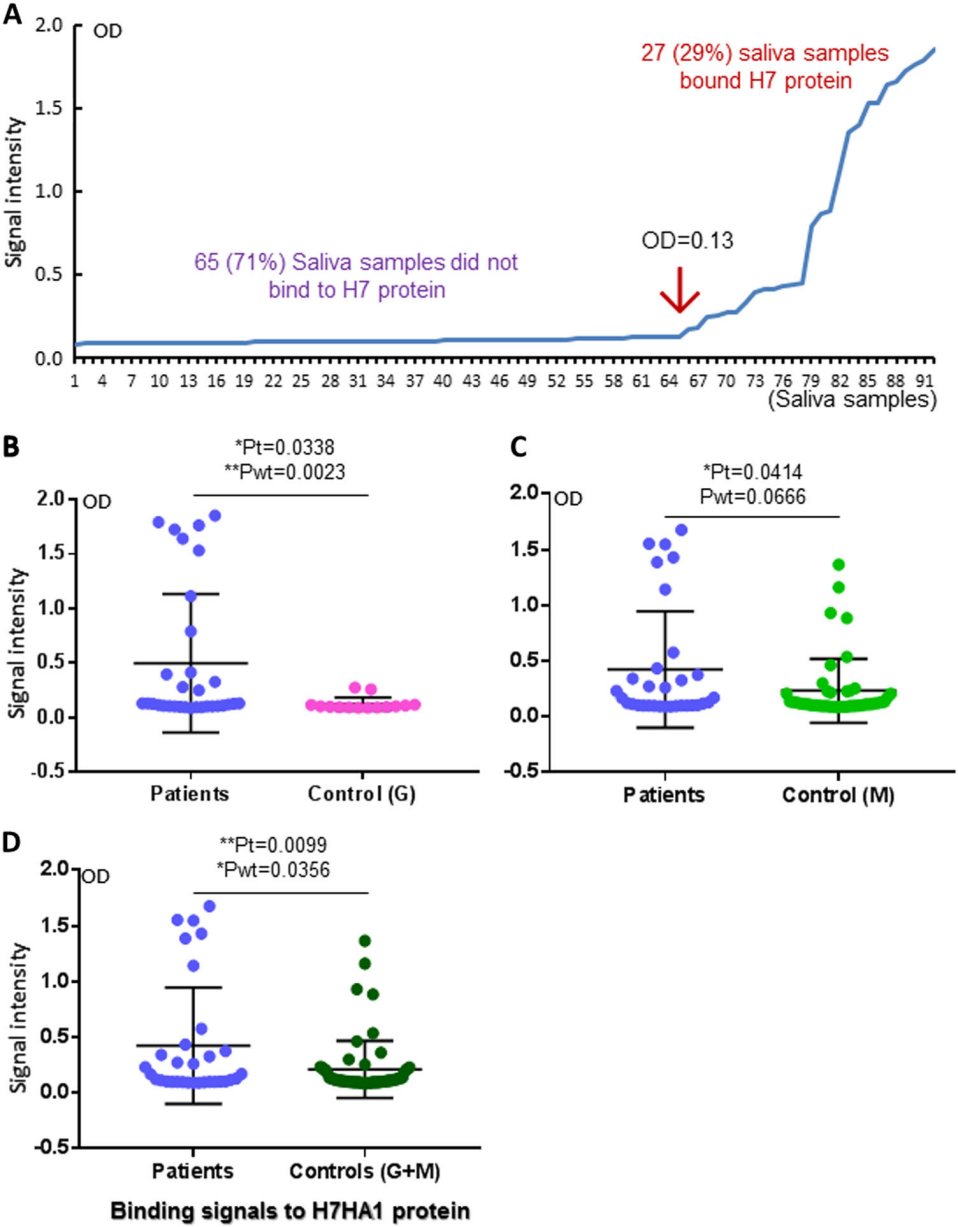
Associations between the S_60_-HA1 protein-binding signals of the saliva samples and H7N9 virus infection. **a** Results of EIA-based binding assays measuring the binding signals between the S_60_-HA1 protein and the 92 tested saliva samples, which were sorted by their signal intensities. The S_60_-HA1 particles bound 27 out of 92 (29%) tested saliva samples, but not the remaining 65 (71%) ones, segregating the saliva samples into bound and non-bound subpopulations. **b**–**d** Comparison of the S_60_-H7 HA1 protein-binding signals among the H7N9-infected patients vs. uninfected co-exposure control group G (**b**), uninfected close contact control M (**c**), and both G and M (**d**) as indicated. *Y*-axis indicates signal intensities, while *X*-axis shows the comparison combinations as indicated. The statistical differences between data groups are analyzed by *t*-test (t) as well as Welch’s *t*-test (wt) and their *P* values (Pt and Pwt) are shown and indicated by asterisks (*P *<* 0.05, **P *<* 0.01)

EIA-based binding assays showed that the S_60_-HA1 protein bound 27 (29%) of 92 tested saliva samples with optical density (OD) > 0.13, but not the remaining 65 (71%) ones, segregating the saliva samples into binder and non-binder subpopulations (Fig. 2a, Table 1). Significantly higher binding signals (Fig. 2d) and higher binding percentage (Table 1) of the S_60_-HA1 protein to the saliva samples of the patients than those to the uninfected controls were found, indicating an association between the H7 HA1-saliva-binding signals and human susceptibility to H7N9 avian influenza viruses.

**Table 1:** Salivary SLe^x^ phenotypes and saliva-binding results to the H7 HA1 protein of H7N9-infected patients and uninfected control groups

Groups of saliva samples	Total	Infected patients^a^	Uninfected controls	
			Co-exposure G^b^	Close contact (M)^c^
(*N*)	92	32	14	46
Age group	Adults	Adults	Adults	Adults
Health/symptoms	/	Flu disease	Healthy	Healthy
H7 HA1 binders (%)	27 (29)	13 (41)	2 (14)	12 (26)
SLe^x^ positive (%)	46 (50)	19 (59)	8 (57)	19 (41)

^a^These were confirmed flu patients infected by H7N9 influenza viruses

^b^These were uninfected control individuals who had similar avian exposure as that of the patients

^c^These were uninfected control individuals who had close contact with the patients

The association became even clearer when the two uninfected controls, each representing the healthy individuals who had similar avian exposure of the patients (control G) and those family members or friends who had close contact with the patients (control M) (see Materials and methods for more details), were analyzed separately (Fig. 2b, c). Specifically, all 12 high binders of the patient group to the S_60_-HA1 protein with OD > 0.275 were infected by H7N9 viruses (Fig. 2b, left panel), but none of the two low and 12 non-binders of the control G group were infected (Fig. 2b, right panel), although the remaining 20 patients were low or non-binders (Fig. 2b, left panel). Noteworthy, while the overall binding signals of the control M were significantly lower than those of the patients (Fig. 2c), the S_60_-HA1 high binders vs. low and no binders were more evenly distributed between the two groups compared with those between the patients and the control G group, suggesting similar genetic and glycan makeups among family members and implying a low efficiency of human-to-human transmission of the H7N9 viruses.

### Salivary SLe^x^ signals were associated with H7N9 virus infection in humans

**Fig. 3:**
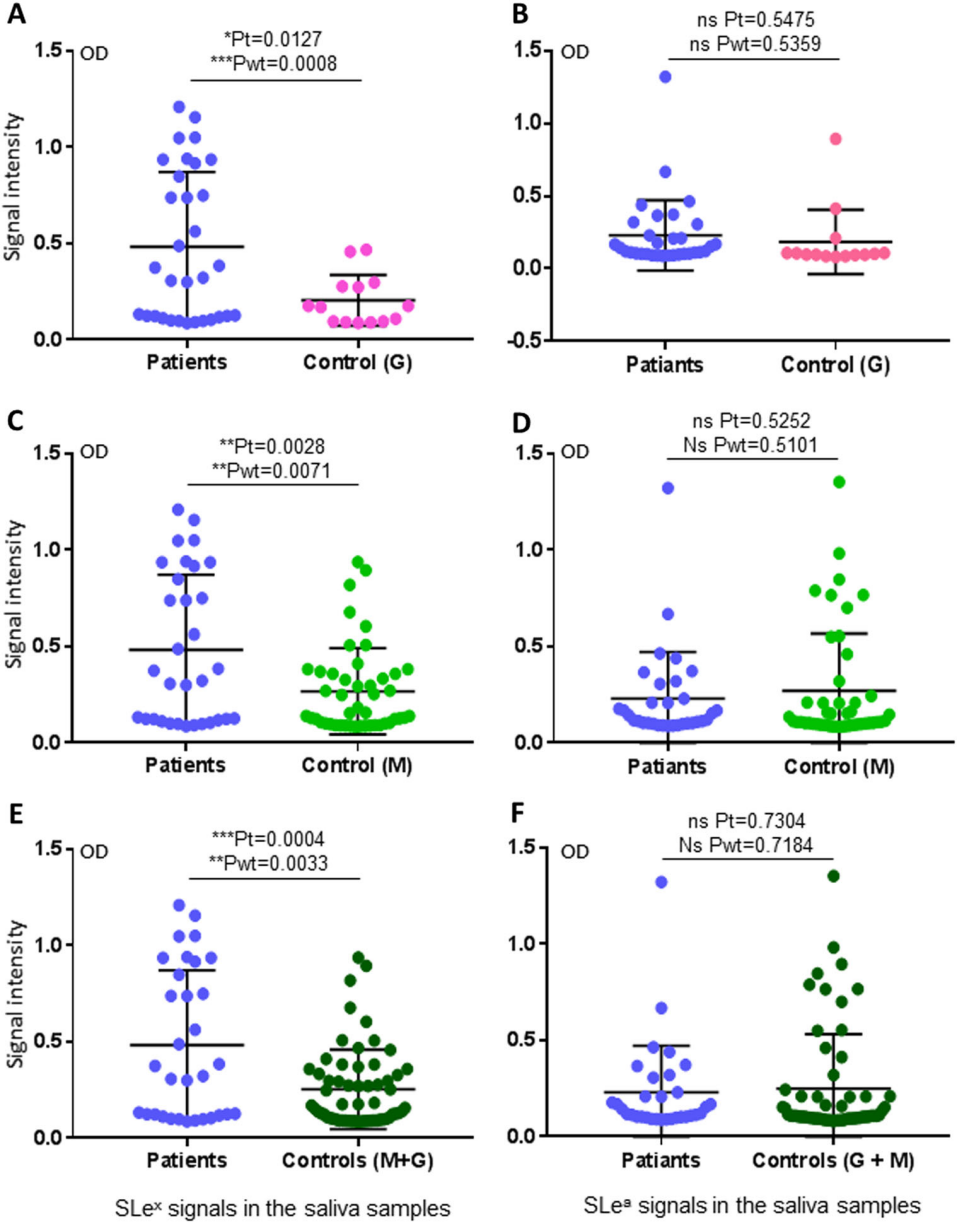
Assessments of associations between the salivary SLe^x^/SLe^a^ phenotypes and H7N9 virus infection. (Left panel) Comparisons of the SLe^x^ phenotypes between the patients and the two uninfected controls in three combinations: **a** patients vs. control G; **c** patients vs. control M; **e** patients vs. controls G + M. (Right panel) Comparisons of the SLe^a^ phenotypes between the patients and the two uninfected controls in three combinations as indicated: **b** patients vs. control G; **d** patients vs. control M; **f** patients vs. controls G + M. *Y*-axis indicates signal intensities, while *X*-axis showed the compared experimental groups. The statistical differences between data groups are analyzed by *t*-test (*t*) as well as Welch’s *t*-test (wt) and their *P* values (Pt and Pwt) are shown and indicated by asterisks (ns, *P* > 0.05; *P < 0.05; **P < 0.01; ***P < 0.001)

We then performed HBGA phenotyping of the saliva samples for glycan factors that are possibly associated with H7N9 virus infection. Among the nine major ABH and Lewis HBGAs tested, only the signals of SLe^x^ antigens were found to be associated with H7N9 infection in humans (Fig. 3a, c, e). By contrast, no such association was found for the other eight HBGAs, including A, B, H1, Le^a^, Le^b^, Le^x^, and Le^y^, as well as SLe^a^ (Fig. 3b, d, f, Supplementary Figures S1 and S2). These data indicated that SLe^x^ antigens may be a host susceptibility factor of avian H7N9 virus to humans. However, when the SLe^x^ antigen signals of the saliva samples were compared with the binding signal profile of H7 HA1, no association between the two sets of data were seen; specifically, 46 (50%) saliva samples are SLe^x^-positive (Fig. 3e, Table 1), while only 27 (29%) saliva samples bound H7N9 HA1 (Fig. 2d, Table 1). These data suggested that while SLe^x^ is an important ligand recognized by HA1 of H7N9 virus, additional unknown saccharide residues adjacent to the SLe^x^ moiety may also affect the binding affinity and specificity of H7N9 HA.

### *Sambucus nigra* lectin- but not *Maackia amurensis* lectin I-binding signals were associated with H7N9 virus infection in humans

**Fig. 4:**
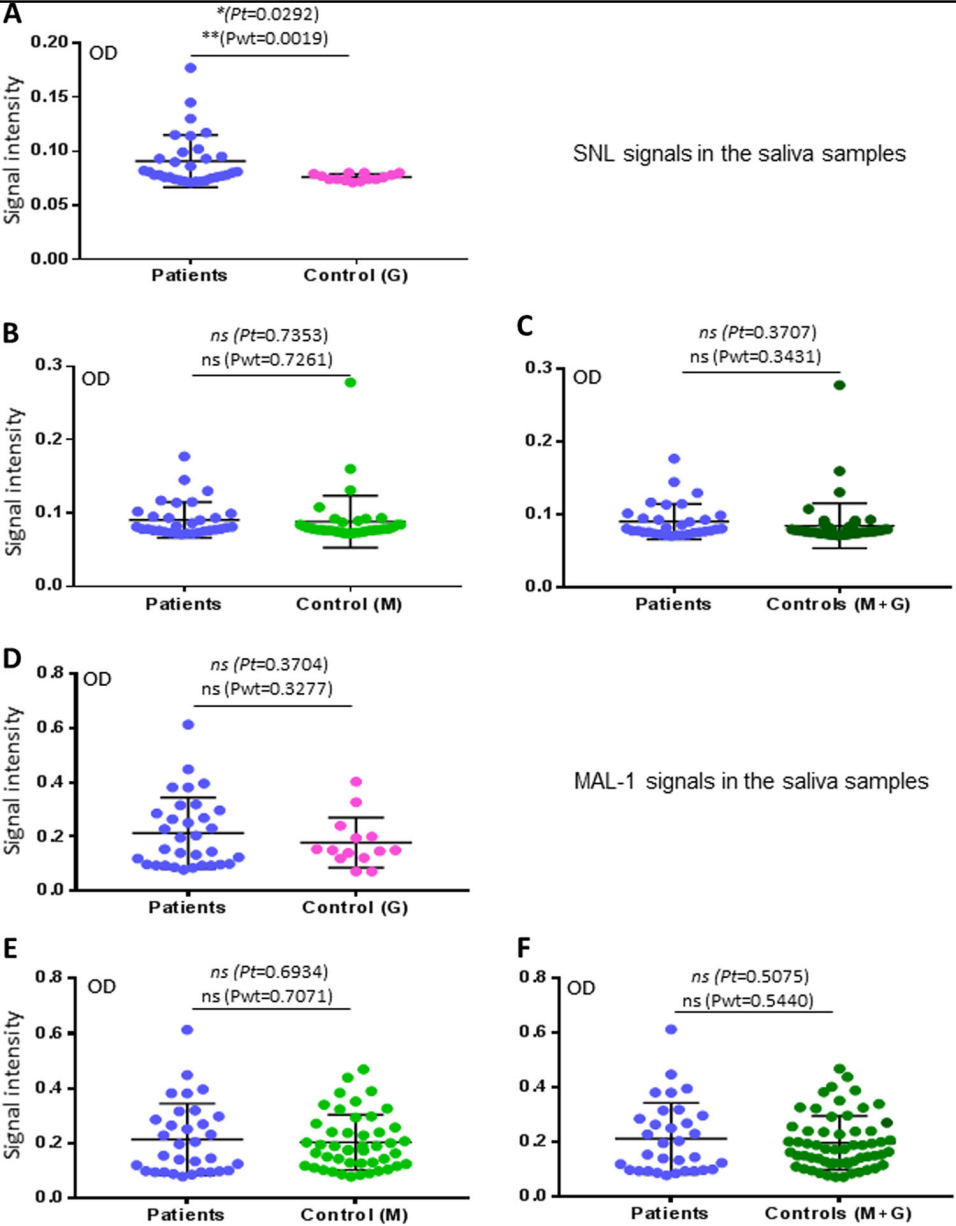
Assessments of associations between the SNL- and MAL I-binding signals to saliva samples and H7N9 infection. **a**–**c** Comparisons of the SNL-binding signals to the saliva samples between the patients and the two uninfected controls in three combinations: **a** patients vs. control G; **b** patients vs. control M; **c** patients vs. controls G + M. **d**–**f** Comparisons of the MAL I-binding signals to the saliva samples between the patients and the two uninfected controls in three combinations: **d** patients vs. control G; **e** patients vs. control M; **f** patients vs. controls G + M. *Y*-axis indicates signal intensities, while *X*-axis showed the compared experimental groups. The statistical differences between data groups are analyzed by *t*-test (*t*) as well as Welch’s *t*-test (wt) and their *P* values (Pt and Pwt) are shown and indicated by asterisks (*P < 0.05; ns, *P* > 0.05)

We also determined saliva-binding profiles of *Sambucus nigra* lectin (SNL) and *Maackia amurensis* lectin I (MAL I). These two lectins are known to bind different subsets of sialyl glycans. Interestingly, although SNL bound the saliva samples weakly with OD < 0.3 (Fig. 4a–c), its binding signals were found to be associated with H7N9 virus infection, when the co-exposure control G group was compared (Fig. 4a). On the other hand, while MAL I revealed much stronger binding signals to the saliva samples, no association with H7N9 virus infection were found (Fig. 4d–f). These data suggested that only limited portion or moiety of sialyl glycans affect the host susceptibility of the avian H7N9 virus infection in humans, consisting with the notion that SLe^x^ is involved in binding of the H7N9 viruses.

## Discussion

Saliva samples have become a widely used reagent to study pathogen–host interactions for assessment of antipathogen activity and host susceptibility of pathogens that recognize salivary carbohydrates as ligands or receptors, including norovirus^8–10,16^, rotavirus^11,12^, candida albicans^17^, and influenza viruses^18^. Our current study demonstrated significantly higher H7 HA-binding signals to the saliva samples from the H7N9-infected patients than those from the uninfected controls who either had similar avian exposure as patients (control G) or had close contact with the patients (control M). These data indicated that the saliva-based binding assays may be used to study and even predict human susceptibility to H7N9 infection.

HBGA phenotyping and lectin-binding profiles of the saliva samples also showed associations between H7N9 infection and salivary SLe^x^ phenotypes and SNL-binding signals, consisting with the previous findings that several types of avian influenza viruses recognize certain SAs and SLe^x^ antigens^5,6^. However, not all SA- and/or SLe^x^-positive saliva samples bound H7N9 HA1 proteins, suggesting residues in addition to the SA and/or the SLe^x^ moieties of the host glycans are responsible for H7N9 host specificity and further implying genetic polymorphism in controlling the binding variations in different human populations. Interestingly, the data that none of the 14 saliva samples from the uninfected controls with similar avian exposure history as the patients (control G) had strong H7 HA1-binding signals (Fig. 2b) and that similar high rates of binders were seen between patients and close contact/family members (Fig. 2c) supported such genetic polymorphism and application of the saliva-HA-binding assay to assess human susceptibility to H7N9 infection.

We also noted that saliva samples of 20 H7N9 virus-infected patients did not bind H7N9 HA (Fig. 2b, Table 1), suggesting that additional avian or human flu strains that recognize different subsets of glycan receptors co-circulated in the same season and infected some of these patients, leading to the confused data. Future studies to exclude these possibilities are necessary. The current study was initiated in the middle of the 2016–2017 epidemic season with many cases being retrospectively recruited based on previous reported cases during the regular avian flu surveillance process at different local Centers for Disease Control and Prevention (CDCs) around Jiangsu province. All 32 H7N9-infected patients were confirmed by PCR using H7- and N9-encoding gene-specific primers, some of the cases were further confirmed by H7N9 virus isolation and sequencing, but still 13 cases were not yet further confirmed by virus isolation and sequencing. Thus, a prospective surveillance with standardized and stricter diagnosis methods, as well as better designed controls need to be performed in future.

The potential human genetic polymorphism in controlling the SA- and/or SLe^x^-containing glycan makeups and the epidemic features of H7N9 caused infections in avian and different human populations raises a question on H7N9 evolution under selection of host carbohydrate receptors in avian and humans. For example, since both HA1 protein and SLe^x^ monoclonal antibody (Mab) bound SLe^x^ antigen in the oligosaccharide-based binding assays, we speculated that the binding interface of H7N9 viruses may be wider than that of the SLe^x^ Mab. As a result, while the Sle^x^ is a ligand of the two probes, additional residues adjacent to Sle^x^ moiety may participate in the binding and thus determine the binding specificity of H7 HA. This scenario might be similar to the complex binding interfaces of human rotaviruses that recognize structurally related HBGAs with different terminal and internal residues being responsible for strain-specific host ranges as a result of selection by the polymorphic HBGAs in rotavirus evolution^19,20^.

Like rotaviruses, IAVs also have a segmented RNA genome, recognizing carbohydrate receptors, including SAs, and interacting with glycan receptors via the surface protruding proteins (HA). We speculate that the wider binding interface of H7N9 virus might also result from host selection, similar to that of rotaviruses. While majority of avian flu viruses recognizing certain SAs and/or SLe^x^ antigens and maintained well in avian populations, H7N9 viruses could bind additional residues adjacent to SLe^x^, gaining further ability to infect humans. Thus, the small susceptible human population of H7N9 viruses as suggested by our study and the low efficiency of human-to-human transmission suggested by others^21^ together explain why only sporadic cases of H7N9 human infection were seen in each epidemic season with typical bird migration pattern. On the other hand, similar principle of divergent evolution could be applied to the seasonal influenza viruses, which were started from the common ancestor viruses in avian but introduced to human by recognize more popular sialyl glycans in the general population and have much larger host target population compared with the avian influenza viruses. This broad host range allowed the seasonal influenza viruses to maintain in human population through the off season without a need of a specific animal carrier, explaining their widely distribution and large scale of epidemics or pandemics. Future studies to test this hypothesis are warrant.

Our study suggests a possible new thinking about the strategy of disease control and prevention against H7N9 flu epidemics. For example, workers who need to expose to avian, such as those working in chicken farms and chicken product industries should be screened using saliva samples and the H7 HA and/or the other two probes for their susceptibility to the viruses and avoid such positions, if they are high binders. The screen strategy may also be applied to the general populations for self-protection purposes to avoid avian exposure. In addition, our study may also help risk assessment on whether a large-scale killing of chicken/ducks as risk control methods may be necessary. Finally, our study suggested that the chance of avian influenza virus-caused large outbreaks or pandemics might be lower than our previously thought, although further studies are necessary to prove these hypotheses.

## Materials and methods

### Patient definition

Avian flu cases occurred in 2016–2017 winter season were reported from local hospitals to CDC of Jiangsu Province. All 32 patients involved in this study met following criteria: (1) had typical clinical symptoms of the flu; and (2) were positive for avian H7N9 flu viruses shown by reverse transcription-PCR (see below). A subset (*n* = 19) of the patients were further confirmed by laboratory isolation of H7N9 flu viruses followed by H7- and N9-encoding gene sequencing (*n* = 13). H7N9 virus infection was shown by real-time PCR assay with H7N9 detection kit (Jiangsu Bioperfectus Technologies Co., LTD, Taizhou, China). All 13 isolated viruses that were sequenced were confirmed as H7N9 viruses. The resulting sequences have been deposited in the public database GISAID (https://www.gisaid.org/) with accession numbers EPI887853–EPI887860 (A/Jiangsu/60467/2016); EPI887893–EPI887900 (A/Jiangsu/60460/2016); EPI1103912–EPI1103919 (A/Jiangsu/06455/2016); EPI1103816–EPI1103823 (A/Jiangsu/08167/2016); EPI1103824–EPI1103831 (A/Jiangsu/08173/2016); EPI1103228, EPI1103229, EPI1103231, EPI1103232, EPI1103234, EPI1103236, EPI1103237, and EPI1103239 (A/Jiangsu/08190/2017); EPI1103252–EPI1103257, EPI1103259, and EPI1103260 (A/Jiangsu/08192/2017); EPI1103294–EPI1103298, EPI1103300, EPI1103301, and EPI1103304 (A/Jiangsu/08197/2017); EPI971205–EPI971212 (A/Jiangsu/11586/2017); EPI971197–EPI971204 (A/Jiangsu/11588/2017); EPI971173–EPI971180 (A/Jiangsu/11589/2017); EPI1103185, EPI1103187–EPI1103190, EPI1103192, EPI1103193, and EPI1103195 (A/Jiangsu/08186/2016); EPI1103365, EPI1103366, EPI1103368–EPI1103370, and EPI1103372–EPI1103374 (A/Jiangsu/11582/2017).

### Study design and saliva sample collection and grouping

To study the transmission of avian flu in humans, saliva samples from all patients and two groups of controls were collected. Of the two control groups, one (*n* = 46) consisted of family members of patients, who had close contact with the patients by providing care to, living with, and/or exposing directly to respiratory secretions or body fluids of the patients during the 14 days immediately before illness onset. The other group (*n* = 14) consisted of healthy individuals with heavy exposure to avian, including those working in chicken farms, chicken markets, and chicken slaughters/processors, who had similar exposure history as their co-worker patients but were not infected.

The saliva samples were collected by Jiangsu Provincial CDC. According to the law of China, data collection on patients was the part of the continuing public health outbreak investigation. Thus, informed consent was waived. The institutional review board of Jiangsu Provincial CDC approved the assessment of the saliva samples from control individuals of the close contact group and the co-exposure group. Written informed consents were obtained from these saliva donors.

### Saliva sample preparation

The saliva samples were prepared according to our previous study^22^. Briefly, the saliva samples were boiled for 10 min to inactivate protein components, including antibodies. The boiled samples were then centrifuged to remove unwanted cells/debris by centrifugation at 10 000 rpm for 5 min on a bench-top centrifuge. The samples were store at −70 °C. The saliva samples were diluted 1000-folds before they were coated to microtiter plates.

### Production of H7 HA1 protein

The receptor-binding HA1 head (223 residues, spanning from C32 to N254) of the H7 protein of an H7N9 virus isolate A/Jiangsu/60462/2016 that infected the patients of this study was produced via the *E. coli* system in two forms, a GST-H7 HA1 fusion protein and a norovirus S_60_ particle (M.T. and X.J., manuscript is under revision) presented HA1 protein (referred as S_60_-H7 HA1). For the GST-H7 HA1 fusion protein, the H7 HA1 protein was fused directly to the GST tag using the plasmid pGEX-4T-1 of the GST-Gene Fusion System (GE Healthcare Life Sciences). For the S_60_-H7 HA1 protein particle, the H7 HA1 protein was fused to the modified norovirus S domain via a short linker (M.T. and X.J., unpublished data). A Hisx6 tag was fused to the end of the H7 HA1 for purification purpose. Both proteins were expressed in *E. coli* as described elsewhere^23,24^.

### Reactivity of the H7 HA1 proteins with H7 antibody

The serially diluted native and boiled (for 5 min) GST-HA1 and S60-HA1 proteins at indicated concentrations, as well as their negative controls (GST/S_60_ particles), were coated, respectively, on Immulon high binding polystyrene microtiter plates (Thermo Scientific) at 4 °C for 16 h. After blocking with 5% nonfat milk for 60 min, rabbit antibody against IAV H7 HA (aa19–524, LifeSpan BioSciences, Inc) at 1:1000 dilution was incubated with the coated protein. The bound antibody was detected by goat-anti-rabbit IgG-horse radish peroxidase (HRP) conjugates (ThermoFisher Scientific) at 1:2000 dilution.

### Hemagglutination

Human RBCs that were provided by the Translational Core Laboratory, Cincinnati Children’s Hospital Medical Center and chicken blood that was provided by local farmers were used for hemagglutination assays, as described elsewhere^25^. Briefly, RBCs were diluted in 0.01 M phosphate-buffered saline (PBS, pH 7.2) without Ca^2+^ or Mg^2+^ (Invitrogen, Carlsbad, CA) and centrifuged for 15 min at 500 × *g*. Five hundred microliters of 0.5% RBCs in 0.01 M PBS (pH 7.2) with serially diluted GST-HA1 or S60-HA1 proteins were mixed in 96-well V-bottom plates and incubated for 60–100 min at 4–8 °C. Agglutinations were observed and photographed, they were also examined by microscopy. The S_60_ particles and GST without HA1 proteins were used as negative controls.

### H7 HA1 protein binding to human saliva samples

All collected saliva samples were boiled for 10 min to inactivate protein components. The boiled saliva samples were then diluted 1000-folds in 1× phosphate buffer saline (PBS, pH 7.4) and coated on Immulon high binding polystyrene microtiter plates (Thermo Scientific) at 4 °C for 16 h or up to 3 days. For solid-phase EIAs, each saliva-coated well of the microtiter plate was blocked with 200 µl 5% nonfat milk for 60 min, followed by adding 100 µl recombinant S_60_-HA1 protein (5 ng/µl in 1× PBS) and incubated for 60 min. The bound S_60_-HA1 proteins were detected by in-house-made guinea pig hyperimmune serum against norovirus-like particles (VLPs)^26^ that recognize the S_60_ particles. The bound Guinea pig antibody was then detected by rabbit anti-guinea pig IgG-HRP conjugate as described elsewhere^23,27^. Five percent nonfat milk in 1× PBS was used as diluent for H7 HA1 protein and the antibodies. Plates were washed five times between above steps using washing solution with 0.05% Tween 20. Wells with 5% nonfat milk without a saliva sample were used as negative controls.

### HBGA phenotyping

The ABH and Lewis HBGAs in the saliva samples were determined by EIAs using corresponding Mabs against individual HBGAs (A, B, H1, Le^a^, Le^b^, Le^x^, Le^y^, SLe^a^, and SLe^x^, purchased from Invitrogen and Biolengend) as described previously^22,26^. Briefly, saliva-coated microtiter plates (Immulon high binding polystyrene microtiter plates, Thermo Scientific) were blocked with 5% nonfat milk, followed by incubation with Mabs against individual HBGAs. The bound Mabs were then detected by a secondary antibody-HRP conjugates. Five percent nonfat milk in 1× PBS was used as diluent for the antibodies. Plates were washed five times between above steps using washing solution with 0.05% Tween 20. The bound signals were displayed by adding HRP substrate reagents. Four well-characterized saliva samples containing all interested HBGAs were included in each microplate as internal controls. Wells with 5% nonfat milk without a saliva sample were used as negative controls. The signal intensity was measured at OD_450._

### EIAs for lectin–saliva interactions

EIA-based assays to measure two lectins, SNL and MAL I (Vector Laboratories), binding to saliva samples were performed via a similar procedures as described above. Saliva samples were coated on microtiter plates (Immulon high binding polystyrene microtiter plates, Thermo Scientific). After blocking with 5% nonfat milk biotinylated SNL and MAL I at 10 µg/ml were added to saliva-coated wells separately and incubated for 60 min. The bound SNL or MAL I was detected by streptavidin-HRP conjugates (Jackson ImmunoResearch, West Grove, PA). The signal intensity was measured at OD_450_.

### EIAs for H7 HA1 protein/lectin–oligosaccharide interactions

To measure interaction between H7 HA1 protein and oligosaccharides, various oligosaccharides (GlycoTech, Rockville, MD) at 2 µg/ml were coated on microtiter plates (Immulon high binding polystyrene microtiter plates, Thermo Scientific) at 4 °C overnight. After blocking with 5% nonfat milk, the plates were incubated with GST-HA1 or S_60_-HA1 protein (10 ng/µl in 1× PBS) for 60 min. The bound GST-HA1 or S_60_-HA1 protein proteins were detected by our in-house-made rabbit hyperimmune serum against GST or guinea pig hyperimmune serum against norovirus VLPs that recognize the S_60_ particles, followed by the detection of rabbit/guinea pig antibodies using specific secondary antibody-HRP conjugates, as described above.

### Data presentation, graphics, and statistical analysis

Productions of H7 HA1 proteins were shown by sodium dodecyl sulfate polyacrylamide gel electrophoresis gel, while binding signals of the H7 HA1 protein to sialyl glycans were shown by column graphics with indications of means (columns) and corresponding standard deviations (error bars) (Fig. 1). Binding and non-binding populations of all tested saliva samples were shown by binding a curve in the order of binding signals from low to high with an indication of the starting of the binding signal by an arrow (Fig. 2a). All remaining data were shown by grouped point graphics, in which each data point represents a single saliva sample that were a mean of triplicated or duplicated experiments. The means (the middle wider lines) and the error bars (the upper and lower narrow lines) for all data points were shown. Statistical differences among data sets were calculated by softwares GraphPad Prism 6 (GraphPad Software, Inc). We first performed unpaired *t*-tests and *F*-tests simultaneously between data sets through the Graphpad software. In cases that *F*-test showed unequal variances between the analyzed data sets, the data sets will be reanalyzed using Welch *t*-tests (unpaired *t*-test with Welch’s correction). In these cases, both *t*-test *P* values are shown. *P* values were set at 0.05 (*P* < 0.05) for significant difference, 0.01 (*P* < 0.01) for highly significant difference, and 0.001 (*P* < 0.001) for extremely significant difference.

## Electronic supplementary material

Supplemental figure S1

Supplemental figure S2
