# Supplementary figures and images for: Saliva as a source of reagent to study human susceptibility to avian influenza H7N9 virus infection
Source: Emerg Microbes Infect. 2018 Sep 19;7:156. doi: 10.1038/s41426-018-0160-8 (PMC6143562; doi:10.1038/s41426-018-0160-8)

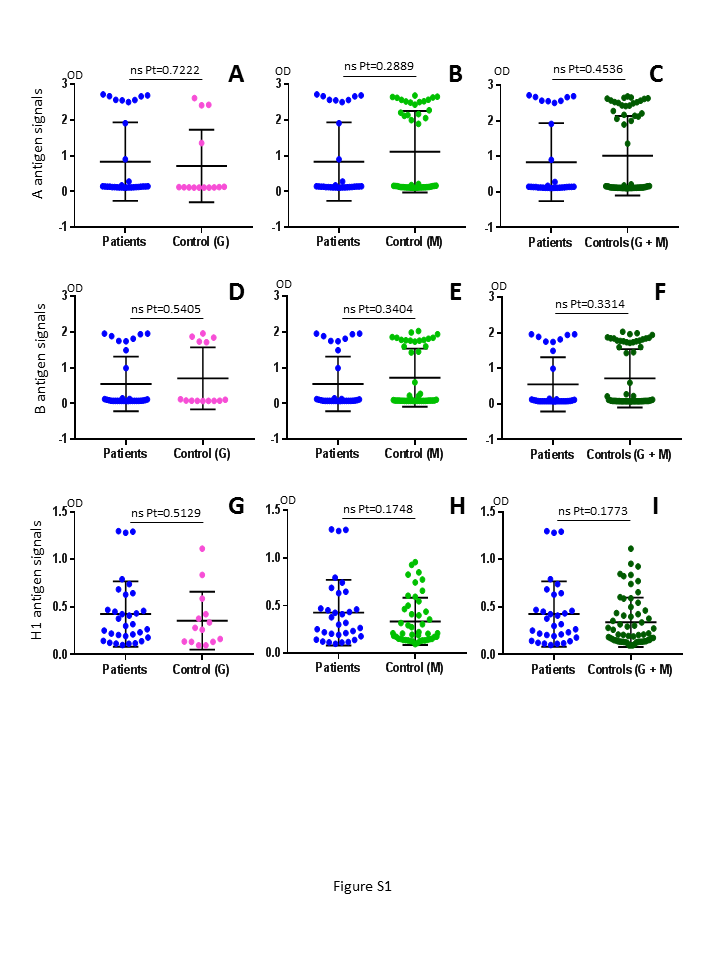

Supplement: Supplementary file 1 — Supplemental figure S1 [file 41426_2018_160_MOESM1_ESM.tif]

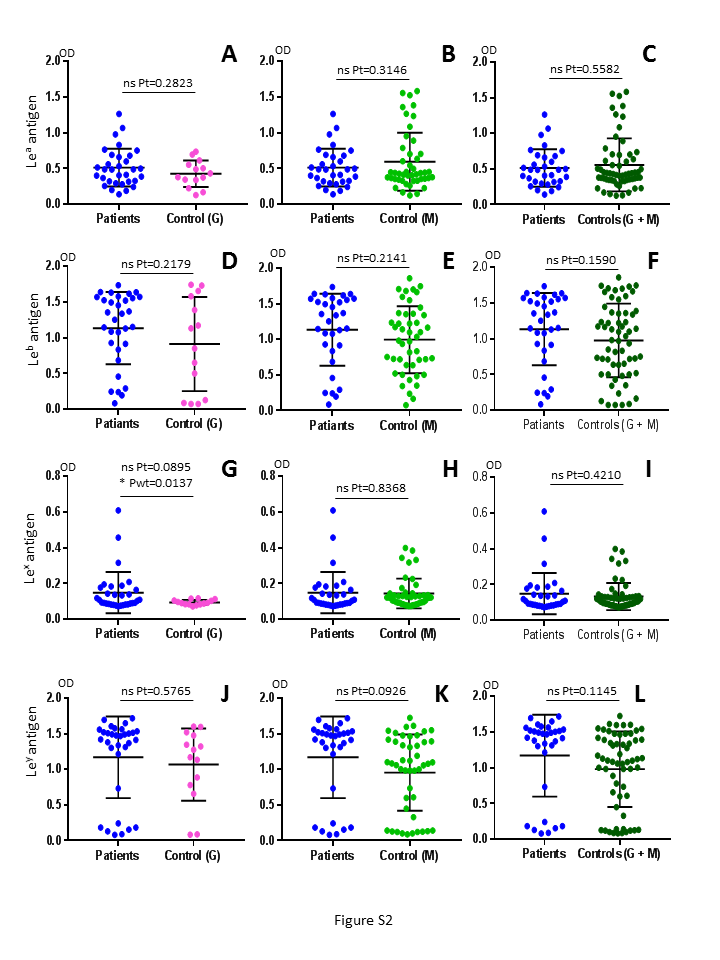

Supplement: Supplementary file 2 — Supplemental figure S2 [file 41426_2018_160_MOESM2_ESM.tif]
